# Second bone marrow transplantation into regenerating hematopoiesis enhances reconstitution of immune system

**DOI:** 10.3389/fimmu.2024.1405210

**Published:** 2024-06-14

**Authors:** Kateřina Faltusová, Martin Báječný, Tomáš Heizer, Petr Páral, Chia-Ling Chen, Katarína Szikszai, Pavel Klener, Emanuel Nečas

**Affiliations:** Institute of Pathological Physiology, First Faculty of Medicine, Charles University, Prague, Czechia

**Keywords:** stem cell, transplantation, hematopoiesis, regeneration, immune system, B cells, T cells

## Abstract

In bone marrow transplantation (BMT), hematopoiesis-reconstituting cells are introduced following myeloablative treatment, which eradicates existing hematopoietic cells and disrupts stroma within the hematopoietic tissue. Both hematopoietic cells and stroma then undergo regeneration. Our study compares the outcomes of a second BMT administered to mice shortly after myeloablative treatment and the first BMT, with those of a second BMT administered to mice experiencing robust hematopoietic regeneration after the initial transplant. We evaluated the efficacy of the second BMT in terms of engraftment efficiency, types of generated blood cells, and longevity of function. Our findings show that regenerating hematopoiesis readily accommodates newly transplanted stem cells, including those endowed with a robust capacity for generating B and T cells. Importantly, our investigation uncovered a window for preferential engraftment of transplanted stem cells coinciding with the resumption of blood cell production. Repeated BMT could intensify hematopoiesis reconstitution and enable therapeutic administration of genetically modified autologous stem cells.

## 1 Introduction

The foundational principles of bone marrow and blood cell transplantation (BMT), a potent remedy for numerous severe diseases, are rooted in experimental hematology. Pioneering work by Brecher and Cronkite (1) revealed that cross-circulation between rats with normal and X-ray-damaged hematopoiesis could restore the damaged hematopoietic system. Ford et al. (2) further substantiated this by transplanting murine spleen cells, marked with the T6 chromosomal translocation, or rat bone marrow cells into irradiated mice, thereby providing irrefutable evidence for the reconstitution of hematopoiesis by transplanted cells. The visualization of hematopoiesis regeneration from transplanted cells in the spleen by Till and McCulloch (3) offered insights into the clonal nature of hematopoiesis regeneration from pluripotent cells (4). These seminal studies paved the way for successful therapeutic BMT, a culmination of significant experimental and clinical research endeavors (5–7).

Mouse-based experimental research has identified hematopoiesis-reconstituting cells as a diverse group with varying longevity and lineage specification in blood cell production. Some transplanted cells serve as an early, albeit transient, source of blood cells, while others contribute to blood cell production over a more extended period. A subset of these cells, the hematopoietic stem cells (HSCs), can produce blood cells long-term and have the capacity to restore damaged hematopoiesis following multiple transplantations (8–13). This transient and long-term blood cell production from transplanted cells has also been observed in patients undergoing BMT-based gene therapy (14).

HSCs exhibit heterogeneity in terms of blood cell lineage specification. While some HSCs can generate all types of myeloid and lymphoid cells in a balanced manner, others show a bias towards the preferential production of either myeloid or lymphoid cells. A subset of HSCs is restricted to producing only myeloid or lymphoid cells (8, 10, 11, 15, 16). The developmental potential of HSCs may be even more limited, restricted to lymphoid and granulocyte-macrophage lineages (17), megakaryocyte-erythroid development (10), or T cells (18). HSCs establish complex interactions with various stroma cells, which support their population maintenance while regulating their differentiation (19, 20).

Transplanted HSCs engage in quantitative competition with the host’s HSCs cells (21, 22), and their functionality is significantly influenced by the environment into which they engraft (23). Myeloablative treatments, used to deplete hematopoietic cells in BMT recipients, also inflict damage on the stroma of the hematopoietic tissues (24–26). Hematopoiesis and stroma then regenerate through mutual interactions (24, 25, 27–29). The pre-transplantation treatment-induced damage to hematopoietic tissues triggers an inflammatory reaction (30, 31), resulting in environmental cues that markedly differ shortly after myeloablative treatment and during active hematopoiesis regeneration.

Early hematopoiesis reconstitution is typified by the expansion of phenotypically altered progenitors and a low number of HSCs (12, 32). Once blood cell production resumes, regenerating hematopoiesis continues to engraft transplanted hematopoiesis-reconstituting cells (32, 33). Recognizing the existing knowledge gap regarding BMT applied to hosts with resumed hematopoiesis, and its potential implications for patient treatment, we examined the outcome of a second BMT delivered either shortly after myeloablative treatment or to recipients with vigorously regenerating hematopoiesis engaged in blood cell production. We analyzed the outcome of the second BMT in terms of its engraftment efficiency, functional longevity, and the types of generated blood cells. Our findings reveal that regenerating hematopoiesis readily and selectively accommodates stem cells with a robust capacity to generate B and T cells.

## 2 Materials and methods

### 2.1 Mice

C57BL/6J (CD45.2), B6.SJL-Ptprca Pepcb/BoyJ (CD45.1), CD45.2/CD45.1 F1 hybrid mice (F1), and C57Bl/6-Tg(UBC-GFP)30Scha/J transgenic mice (UBC-GFP) of both sexes were used. The mice were bred in the Center of Experimental Models of the First Faculty of Medicine, Charles University.

### 2.2 Irradiation

Whole-body irradiation from a ^60^Co source of gamma rays (approximately 0.40 Gy/min) was used.

### 2.3 Regeneration of hematopoiesis after transplantation of syngeneic bone marrow cells

Bone marrow cells from one femur (approximately 29 million cells) was transplanted via the retro-orbital route to mice conditioned with irradiation (9 Gy) 2─3 hours prior. Blood samples were obtained at various time points after transplantation and analyzed by the Auto Hematology Analyser BC-5300 Vet (Mindray, China). The weight of wet spleens was determined. Bone marrow was flushed from femurs into one mL of phosphate buffered saline, and cells were counted by the Auto Hematology Analyser BC-5300Vet (Mindray, China).

### 2.4 Competitive transplantation of normal and regenerated bone marrow

Bone marrow cells from CD45.2 mice, either untreated or transplanted with syngeneic bone marrow from one femur 30, 40, or 60 days prior, were mixed with the same number of bone marrow cells from untreated CD45.1 mice. The cell mixture was transplanted to irradiated F1 hybrid mice, and the proportion of CD45.2 and CD45.1 cells was determined in blood after six months.

### 2.5 Immature hematopoietic cells in bone marrow

Bone marrow cells lacking lineage markers and expressing the Sca-1 antigen and c-Kit receptor (LSK cells), subdivided according to the expression of CD150 and CD48 markers, were determined in normal and regenerating bone marrow. Cells were stained for 30 minutes by fluorescently labeled antibodies on ice in the dark and analyzed by the flow cytometer BD FACSAria IIu (BD Biosciences, USA). All antibodies are listed in **Supplementary Material**.

### 2.6 Sequential transplantation of CD45.2 and CD45.1 bone marrow

Mice (CD45.2, CD45.1, F1, or UBC-GF) conditioned by irradiation were transplanted with bone marrow cells from normal CD45.2 or CD45.1 mice (1^st^ BMT) within 2─3 hours after irradiation. The second transplant (2^nd^ BMT) of bone marrow from congenic CD45.1 or CD45.2 mice was administered after 2 hours and for various days up to 60 days after the 1^st^ BMT.

### 2.7 Analysis of chimeric hematopoiesis in peripheral blood

Blood samples were stained with the anti-CD45.2 and anti-CD45.1 antibodies, and antibodies labeling granulocytes and monocytes (GM cells), B cells (B220 cells), and T cells (CD4 and CD8).

### 2.8 Re-transplantation of chimeric bone marrow

Chimeric bone marrow resulting from two successive BMTs was transplanted to lethally irradiated (9 Gy) CD45.2 or CD45.1 secondary recipients. In one experiment, the chimeric bone marrow from the secondary recipients was transplanted into the tertiary UBC-GFP recipient mice. The peripheral blood was analyzed after four and six months.

### 2.9 Statistical analysis

For multi-group comparisons, one-way ANOVA was used. The Student´s t-test with a two-tailed distribution was used to compare results from two experiments. The significance level is indicated as follows: * p < 0.05, ** p < 0.01, *** p < 0.001. The GraphPad Prism software (GraphPad San Diego, USA) was used.

## 3 Results

### 3.1 Hematopoiesis recovers rapidly after transplantation, but recovery of HSCs is delayed

**Figure 1:**
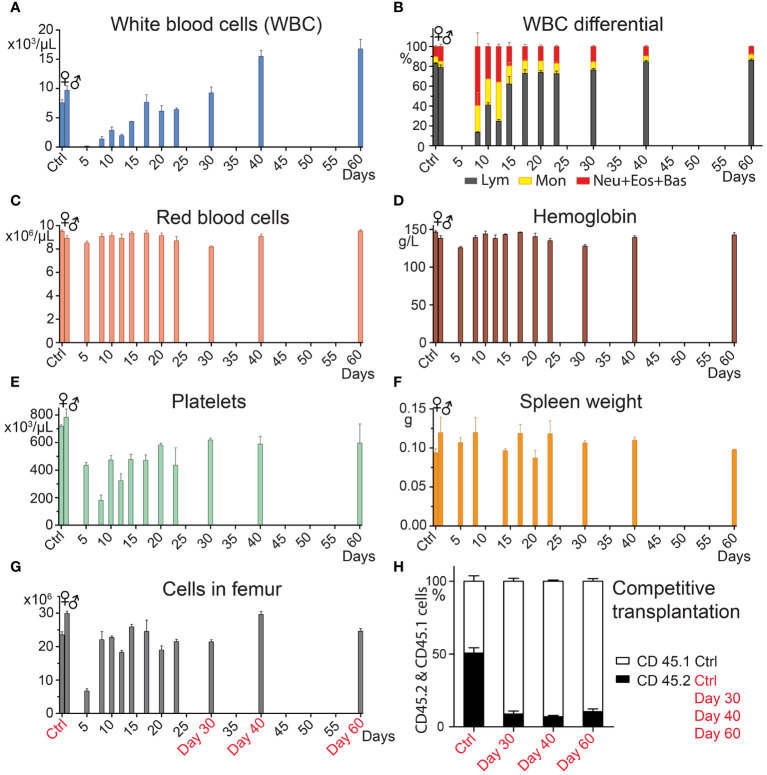
Hematopoiesis recovers vigorously after BMT, but the pool of HSCs remains reduced. In four independent experiments, bone marrow from C57Bl/6 mice was transplanted in amounts corresponding to one femur to groups of three females (days 10─60) and four males (days 5 and 8) 9-Gy irradiated C57Bl/6 mice. Control groups consisted of untreated mice (Ctrl). The transplanted bone marrow was pooled from 5─11 C57Bl/6 donors. Mice were sacrificed 5─60 days after transplantation. **(A–F)** complete blood count and spleen weight. **(G)** bone marrow cellularity in the femur. **(H)** Bone marrow from three untreated CD45.2 mice (Ctrl), and groups of three CD45.2 mice transplanted before 30, 40, and 60 days with bone marrow from one femur were pooled within the groups and mixed with the same number of bone marrow cells obtained from untreated CD45.1 mice. All mice were females. Ten to 12 million cells were transplanted to groups of 5─7 irradiated (7.5 Gy) CD45.2/CD45.1 F1 hybrid mice. After six months, the percentage of the CD45.2 and CD45.1 nucleated blood cells was determined after eliminating the background of CD45.2/CD45.1 cells. Data are means ± SEM.

Clinical BMT aims for early robust recovery of blood cell production by transplanting a high number of hematopoiesis-reconstituting cells, including stem cells and progenitor cells. Before conducting experiments with two transplants applied successively at various periods of hematopoiesis regeneration, we analyzed the time course of hematopoiesis recovery after a single transplant. Robust hematopoiesis regeneration was induced in lethally irradiated mice by transplanting bone marrow cells from one femur. The size of the graft exceeded approximately 200 times the minimum number of cells needed for survival and long-term hematopoiesis reconstitution (13). Bone marrow cellularity significantly increased after day 5 (**Figure 1G**) and spleen weight was normal when determined on day 5 and later (**Figure 1F**). Blood cell production resumed by day10, as shown by the increasing number of white blood cells, red blood cells, and platelets in the peripheral blood (**Figures 1A–E**). Granulocytes and monocytes were mainly produced in the first 12 days, but then lymphocytes also began to be produced in significant quantity (**Figure 1B**). Bone marrow collected 30, 40, and 60 days after BMT was tested for its capacity to compete with normal bone marrow in the reconstitution of damaged hematopoiesis by the competitive transplantation assay. Bone marrow collected 30, 40, and 60 days after BMT produced only 8.9%, 7.0%, and 10.4% cells (**Figure 1H**, Days 30, 40, 60), significantly less than ≈ 50% in mice competitively transplanted with bone marrow from both untreated CD45.2 and CD45.1 mice (**Figure 1H**, Ctrl). Hence, the pool of the cells providing long-term hematopoiesis has only partly recovered 30–60 days after BMT.

The delayed recovery of long-term acting stem cells after BMT corresponds to the slow recovery of immature lineage negative, Sca-1, and c-Kit positive (LSK) cells lacking the CD48 marker (LSK CD48^─^ cells) in the bone marrow (**Supplementary Figure 1**), which contain hematopoiesis-reconstituting cells with middle to long-term functioning after transplantation (8, 32, 34).

Results presented in this section demonstrate the robust recovery of hematopoiesis and blood cell production in the first two weeks after transplantation and the significantly delayed replenishment of the stem cell pool.

### 3.2 Regenerating hematopoiesis prioritizes the engraftment of HSCs

**Figure 2:**
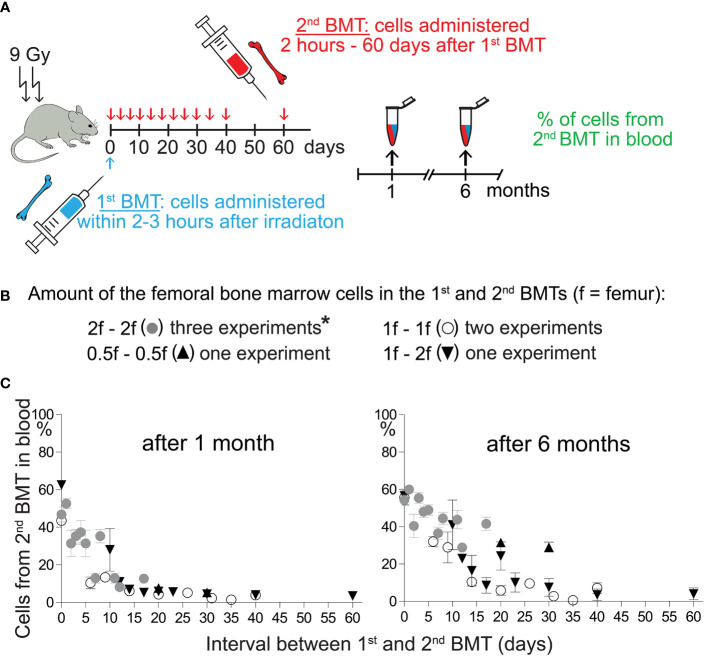
Engraftment of progenitors from the 2^nd^ BMT declines more steeply after the 1^st^ BMT than the engraftment of stem cells. **(A)** Experimental design. CD45.2 or CD45.1 mice were used as the recipients of syngeneic bone marrow cells applied shortly after irradiation (1^st^ BMT). The 2^nd^ BMT was from mice with the opposite CD45 allotype. **(B)** The amount of bone marrow cells administered in the 1^st^ and the 2^nd^ BMT. *These results have been previously published by Báječný et al. (32). **(C)** Results from seven independent experiments showing the percentage of nucleated blood cells derived from the 2^nd^ BMT in the peripheral blood determined one and six months after the 2^nd^ BMT. Results are means ± SEM (n=5**─**8).

Our previous results with split BMT (32, 33) demonstrated the engraftment of stem and progenitor cells during hematopoiesis regeneration and showed a faster decline in the engraftment of progenitors to stem cells. We have verified the previous results in new experiments and show all results in **Figure 2** and **Supplementary Figure 2**.

In the previous and new experiments, regeneration of hematopoiesis was induced in lethally irradiated mice by transplanting syngeneic bone marrow cells corresponding to half, one, or two femurs (1^st^ BMT). Two hours later or after 1–60 days, we challenged the transplanted cells with the second BMT (2^nd^ BMT) from congenic mice in the amount equal (higher in one experiment) cell number (**Figure 2A, B**). **Figure 2C** and **Supplementary Figure 2** summarize results from seven experiments comparing blood cell production from the 2^nd^ BMT after one and six months. The cell production from the 2^nd^ BMT determined after one month declined rapidly after the 1^st^ BMT. The decline was delayed when blood cell production from the 2^nd^ BMT was determined after six months. These results indicate preferential engraftment of HSCs compared to progenitors by regenerating hematopoiesis.

### 3.3 The prioritizing of engraftment of HSCs in regenerating hematopoiesis is highlighted in UBC-GFP recipient mice

Because the results read one month after transplantation could be confounded by the host T cells surviving in the peripheral blood after conditioning irradiation (35), we used UBC-GFP mice as recipients of the two successive BMTs in the next five experiments. The GFP-positive cells of the host were gated out in the peripheral blood analysis of cells derived from the 1^st^ BMT and 2^nd^ BMT. In these experiments, we induced hematopoiesis regeneration in irradiated UBC-GFP mice by transplantation of 20 million bone marrow cells from either CD45.1 or CD45.2 mice (1^st^ BMT). After two hours or after 7–18 days, the mice were transplanted with the same number of bone marrow cells from congenic mice with different CD45 allotype (2^nd^ BMT). The combination of CD45.2 and CD45.1 donors, their sex, and mice numbers in the five experiments are in **Table 1**. One and six months after the 2^nd^ BMT, the impact of the 1^st^ BMT on the outcome of the 2^nd^ BMT was analyzed in the peripheral blood.

**Table 1:** Five independent experiments with two successive BMTs.

Experiment	1	2	3	4	5
Number of twice transplanted mice	5*/4°	3*/3°	3*/4°	4*/4°	4*/4°/4^●^
Donors (20x10^6^ cells) BMT1	CD45.2 male	CD45.1 male	CD45.1 male	CD45.2 male	CD45.2 female
Donors (20x10^6^ cells) BMT2	CD45.1 male	CD45.2 male	CD45.2 male	CD45.1 male	CD45.1 female

* mice that received the 2^nd^ BMT 2 hours after the 1^st^ BMT; °^●^ mice that received the 2^nd^ BMT 7 days (Exp. 1 & 2), 10 days (Exp. 3 & 4), and 13° or 18^●^ days after the 1^st^ BMT.

Each of the five experiments included a group of mice to whom the 2^nd^ BMT was administered 2 hours after the 1^st^ BMT. The aim of the 2-hours experimental groups was testing whether 20 million cells from the first transplant would not acutely reduce the available space for another 20 million cells from the 2^nd^ BMT. The outcome of the 2^nd^ BMT applied 2 hours after the 1^st^ BMT was not affected by the previous BMT when evaluated after one and six months, and CD45.2 and CD45.1 nucleated blood cells were equally represented in all cell types in the peripheral blood (**Supplementary Figure 3**). Therefore, the 1^st^ BMT has not limited the engraftment of the 2^nd^ BMT by occupying a significant portion of the space created for transplanted stem and progenitor cells by conditioning irradiation.

**Figure 3:**
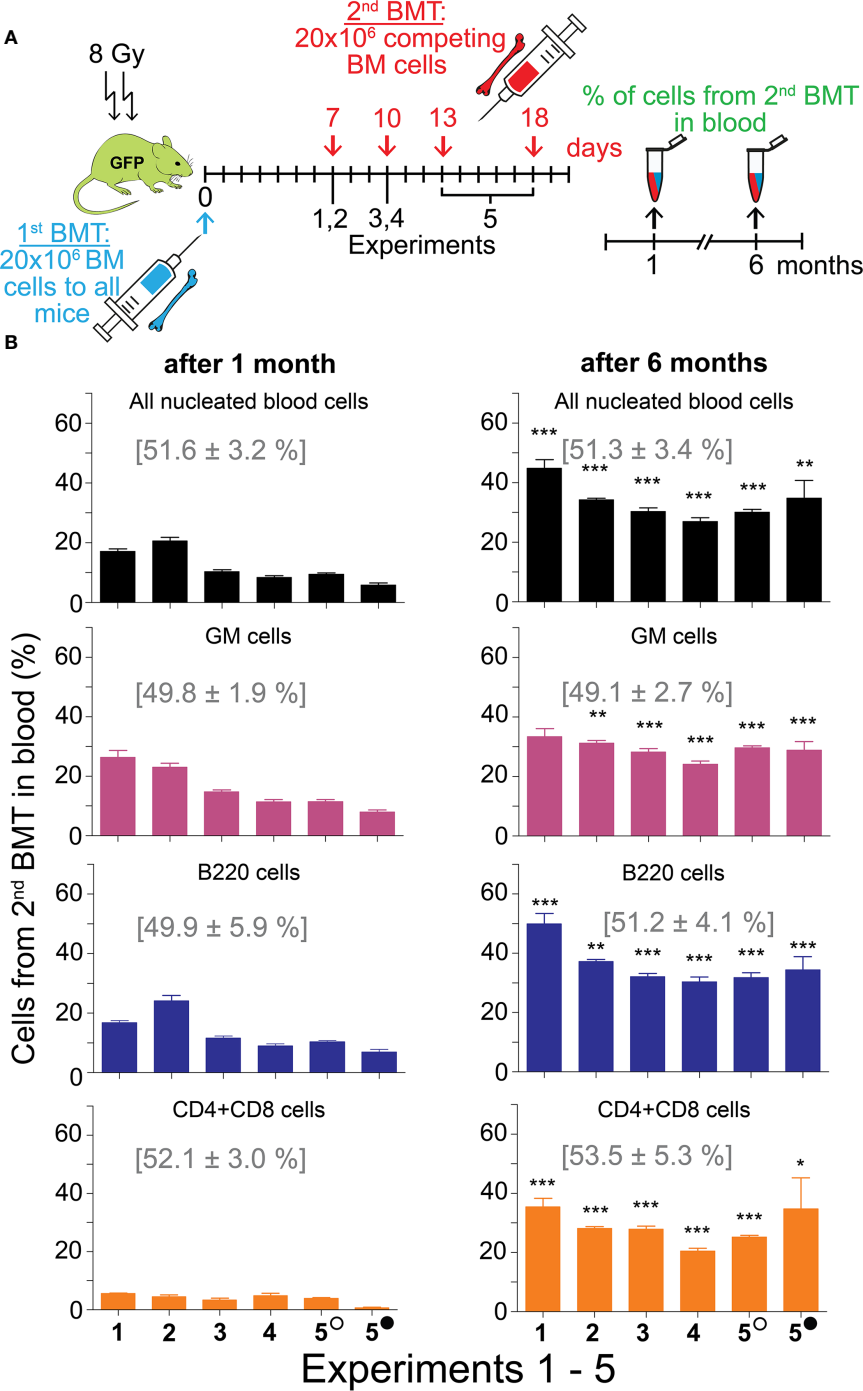
Second BMT administered 7─18 days after the 1^st^ BMT results in significant engraftment of stem cells. **(A)** Experimental design. In five independent experiments, regeneration of hematopoiesis was induced in irradiated UBC-GFP mice by transplantation of 20 million bone marrow cells delivered to all mice in the 1^st^ BMT. After 7 days (Experiments 1 and 2), 10 days (Experiments 3 and 4), or 13 and 18 days (Experiment 5) mice were given the 2^nd^ BMT of 20 million bone marrow cells from congenic mice with the opposite CD45 marker. The combination of CD45 markers, mice sex, and mice numbers in the five experiments are in **Table 1**. A sample of blood was examined for the percentage of nucleated blood cells with the CD45 marker corresponding to the second transplant after one and six months. **(B)** Blood nucleated cells were labeled with antibodies to distinguish granulocytes and monocytes (GM), B cells (B220), and T cells (CD4+CD8). Numbers in brackets show the values in the mice given the 2^nd^ BMT within 2 hours after the 1st BMT (results shown in **Supplementary Figure 3**). Empty circle – 13 days; full circle – 18 days. Results are means ± SEM. *p<0.05, **p<0.01, ***p<0.001 against the values after 1 month.

**Figure 4:**
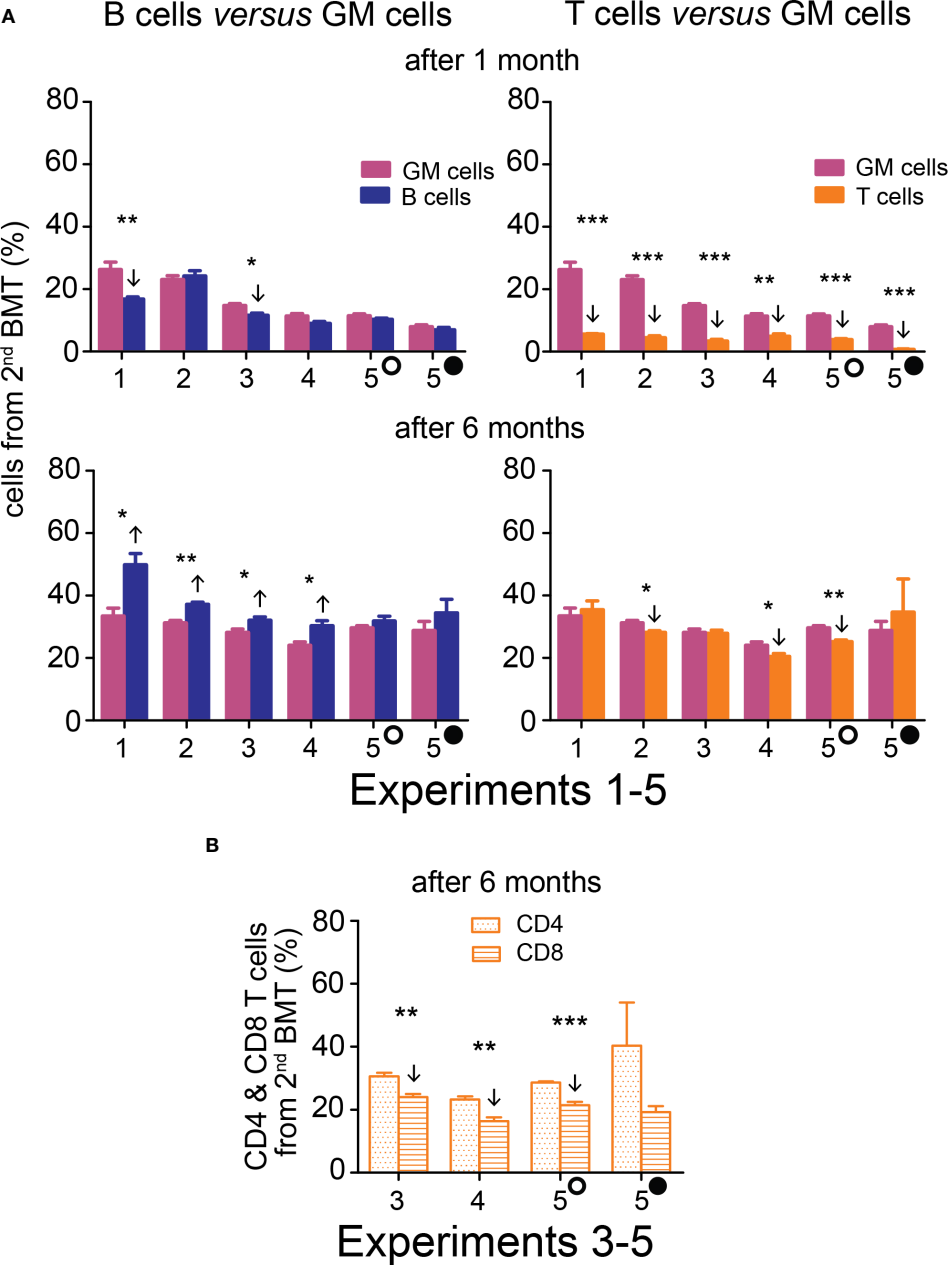
Hematopoiesis derived from the 2^nd^ BMT administered 7─18 days after the 1^st^ BMT is myeloid-biased after one month, but B cell-biased and efficient in T cell production after six months. **(A)** Frequency of B cells (B220) and T cells (CD4+CD8) derived from the 2^nd^ BMT given 7 days (Experiments 1 and 2), 10 days (Experiments 3 and 4), 13 (empty circle) and 18 (full circle) days (Experiment 5) after the 1^st^ BMT is compared to the frequency of granulocyte-monocytes (GM). Results are from the peripheral blood analyzed one and six months after the 2^nd^ BMT. **(B)** In Experiments 3, 4, and 5, CD4 and CD8 T cells were distinguished six months after transplantation. * p < 0.05, ** p < 0.01, *** p < 0.001. Arrows indicate significantly increased or decreased values.

The contribution of the 2^nd^ BMT to nucleated blood cells in the peripheral blood was less than 50% in the five experiments in which the 2^nd^ BMT was administered at intervals of 7 days (Experiments 1 and 2), 10 days (Experiments 3 and 4), and 13 and 18 days (Experiment 5) after the 1^st^ BMT. However, the efficacy of the 2^nd^ BMT was significantly higher when evaluated after six months compared to one month (**Figure 3**; **Supplementary Figure 4**). After one month, cell production from the 2^nd^ BMT was myeloid-biased primarily due to very low production of T cells (CD4 plus CD8) (**Figure 3**; **Supplementary Figure 4**). The production of lymphoid cells (B and T cells; B220 and CD4 plus CD8 cells) is compared to the production of myeloid cells (granulocytes and monocytes; GM cells) in **Figure 4** and **Supplementary Figure 5**. After one month, B cells (B220) and granulocytes and monocytes (GM cells) were produced equally in four out of six groups and less in two groups. After six months, the production of B cells (B220) exceeded the production of GM cells in four out of six groups and was insignificantly higher in two groups. The production of T cells from the 2^nd^ BMT became significant after six months (**Figure 4A**). CD4 and CD8 T cells derived from the 2^nd^ BMT were separately evaluated after six months in Experiments 3–5. CD4 and CD8 were produced equally when the 2^nd^ BMT was administered 2 hours after the 1^st^ BMT (**Supplementary Figure 5**). When the 2^nd^ BMT was administered 10–18 days after the 1^st^ BMT, CD8 T cells were produced less than CD4 T cells (**Figure 4B**).

Results from this section demonstrate that BMT can be effectively administered in separate doses, and regenerating hematopoiesis preferentially engrafts stem cells to the progenitors from the 2^nd^ BMT. HSCs and progenitors from the 2^nd^ BMT rapidly resumed production of B cells and later became a significant source of T cells as well.

### 3.4 Re-transplantation of chimeric bone marrow confirms HSCs engraftment from the 2^nd^ BMT

**Figure 5:**
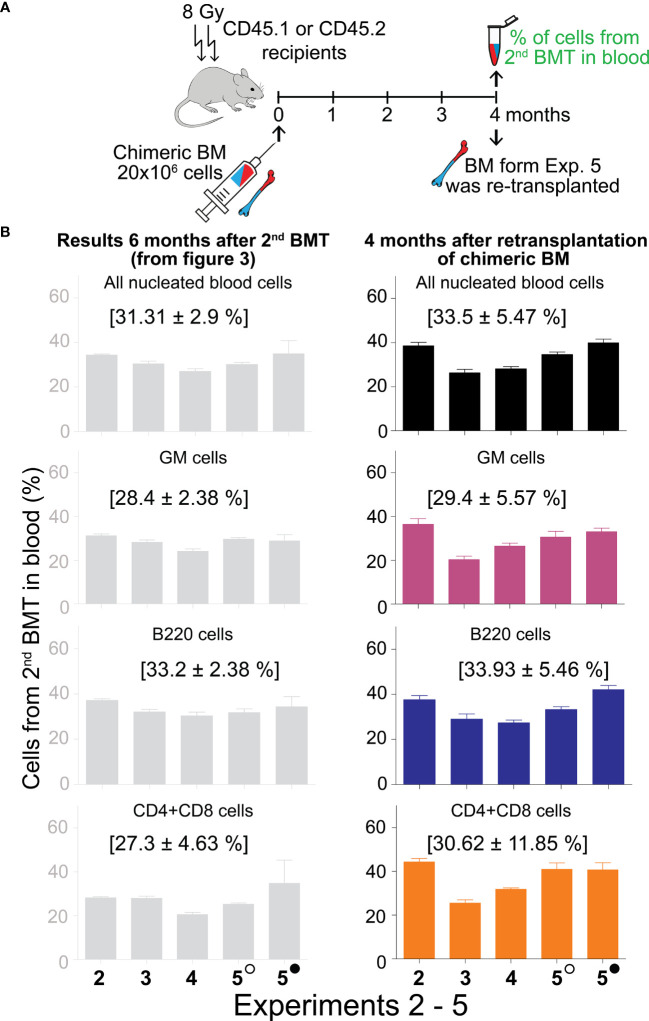
Sustained production of blood cells from the 2^nd^ BMT administered 7─18 days after the 1^st^ BMT in secondary recipients. **(A)** Experimental design. Chimeric bone marrow was collected and pooled from groups of mice to which the 2^nd^ BMT was given 7 days (Experiment 2), 10 days (Experiments 3 and 4), 13 (empty circle), and 18 (full circle) days (Experiment 5) after the 1^st^ BMT. The bone marrow collected six months after two successive BMTs was re-transplanted to secondary 8 Gy irradiated recipients (CD45.2 mice in Experiment 2; CD45.1 mice in Experiments 3, 4, and 5). The peripheral blood cells were analyzed after 4 months. **(B)** The percentage of cells derived from the 2^nd^ BMT given 7–18 days after the 1^st^ BMT is shown in the right column. For comparison, the corresponding results from the donors of chimeric bone marrow (presented previously in **Figure 3**) are shown in the left grey column. In brackets are means ± SEM from Experiments 2–5.

**Figure 6:**
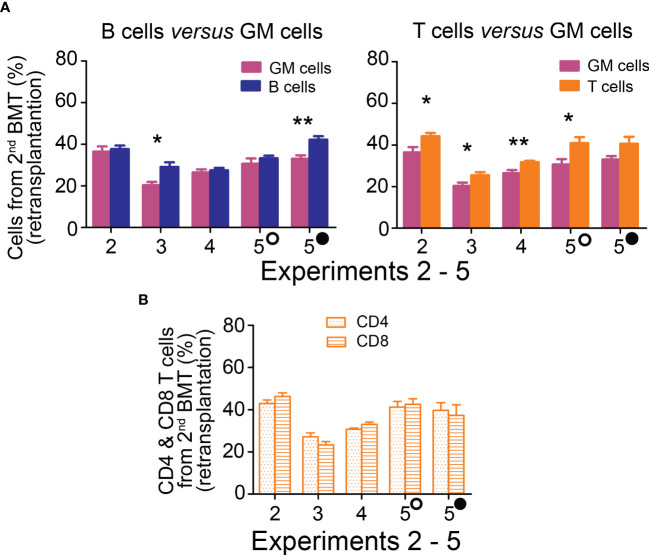
Hematopoiesis derived from the 2^nd^ BMT administered 7─18 days after the 1^st^ BMT is lymphoid-biased in secondary recipients. **(A)** Frequency of lymphoid B cells (B220) and T cells (CD4+CD8) derived from 2^nd^ BMT delivered 7 days (Experiment 2), 10 days (Experiments 3 and 4), 13 (empty circle) and 18 (full circle) days (Experiment 5) after the 1^st^ BMT is compared to the frequency of granulocytes and monocytes (GM) in the peripheral blood four months after chimeric bone marrow transplantation. **(B)** CD4 and CD8 T cells frequency in the peripheral blood. * p < 0.05, ** p < 0.01.

The ultimate indication of HSC stemness is the capacity to reconstitute depleted hematopoiesis over the long term. The longevity assay of HSC functionality involves re-transplantation of reconstituted hematopoiesis to secondary recipients with depleted hematopoiesis, and eventually to tertiary recipients. To conduct these functional tests assessing the durability of HSC pools established from the 2nd BMT, chimeric bone marrow from Experiments 2 to 5 was re-transplanted to secondary recipients. Blood cell production derived from the 2^nd^ BMT was assessed in the peripheral blood after 4 months (**Figure 5B**; colored columns) and compared to that in the primary transplanted mice at the time of bone marrow re-transplantation (**Figure 5B**; gray columns). This demonstrated sustained production of blood cells derived from the 2^nd^ BMT after bone marrow re-transplantation, whether administered 2 hours (**Supplementary Figure 6**) or 7–18 days (**Figure 5**) after the 1^st^ BMT. The BMT administered 7–18 days after the 1^st^ BMT resulted in a higher production of lymphoid cells compared to granulocytes and monocytes in secondary recipients, significantly 6 times and insignificantly 4 times (**Figure 6A**), with the proportion of CD4 and CD8 T cells being equal (**Figure 6B**). The production of lymphoid cells was balanced with the production of GM cells when the 2^nd^ BMT was administered 2 hours after the 1^st^ BMT (**Supplementary Figure 7**).

Chimeric bone marrow from the secondary recipients was re-transplanted to tertiary recipients in Experiment 5, and their peripheral blood was analyzed after four and six months. Blood cell production from the 2^nd^ BMT remained steady from the fourth month after the primary transplantation (**Supplementary Table 1**).

Results from the re-transplantation experiments demonstrated that the 2^nd^ BMT, administered either 2 hours or 7–18 days after the 1^st^ BMT, established durable pools of HSCs which became significant sources of granulocytes and monocytes, as well as B and CD4 and CD8 T cells.

## 4 Discussion

The justification for bone marrow transplantation in hosts with regenerating hematopoiesis is rooted in the minimal presence of HSCs during the early stages of hematopoiesis reconstitution when intensive blood cell production resumes from phenotypically altered progenitors (12, 32).

In our study, the initial BMT of twenty million donor bone marrow cells did not impact the engraftment and efficiency of an additional twenty million cells from the 2^nd^ BMT administered two hours later. Notably, both BMTs, administered within a two-hour interval between each, contributed equally to blood cell production across all examined cell lineages and established identical pools of HSCs that functioned concurrently in the hosts.

The efficacy of the 2^nd^ BMT, administered 7–18 days after the 1^st^ BMT, was significantly reduced when assessed based on blood cell production after one month. However, this reduction was less pronounced when evaluated after six months. This discrepancy aligns with the early expansion of progenitors in regenerating hematopoiesis, which occurs in the presence of a significantly reduced pool of HSCs (12, 32). While the results from the two successive BMTs administered within a 7–18 day period mechanically align with the significant imbalance in progenitor and stem cells, strongly favoring progenitors, it should be recognized that cells from the 2^nd^ BMT, administered 7–18 days after the 1^st^ BMT, are exposed to a markedly different environment in the regenerating hematopoiesis than the cells administered shortly after conditioning irradiation (24, 27–29, 35–38).

A significant finding of our study is the substantial production of lymphoid cells from the 2^nd^ BMT administered to mice with intensely regenerating hematopoiesis. The production of B cells commenced before that of T cells, but both types of lymphoid cells were significantly and sustainably sourced from the 2^nd^ BMT.

The 2^nd^ BMT, administered during the period of intensive hematopoiesis regeneration and resumption of blood cell production, could be applied in therapeutic BMTs when hematopoiesis-reconstituting cells remain available after their initial administration. Cells for the repetitive BMT could potentially be sourced from donor peripheral blood or when cord blood cells are used in patient treatment. Genetically engineered autologous HSCs could be delivered in the 2^nd^ BMT to correct a metabolic or cellular disease (13). The recent advancement in the *ex vivo* expansion of HSCs (39–41), including human HSCs (42, 43), are particularly relevant in this context.

Our research demonstrates that regenerating hematopoiesis readily engrafts HSCs, which subsequently become a potent source of immune cells. Repeated BMT administered after recovery of blood cell production from the initial BMT could potentially reduce the incidence of post-transplantation cytopenia and the risk of graft failure. Patients who are at risk of infections due to pre-transplantation hematopoiesis-reducing therapy could benefit from enhanced reconstitution of the immune system. Repeated BMT could thereby contribute to the safety and effectiveness of this advanced stem cell-based therapy.

## Data availability statement

The raw data supporting the conclusions of this article will be made available by the authors, without undue reservation.

## Ethics statement

The animal study was approved by Animal Welfare Committee of Charles University, First Faculty of Medicine. The study was conducted in accordance with the local legislation and institutional requirements.

## Author contributions

KF: Conceptualization, Data curation, Formal analysis, Visualization, Writing – original draft. MB: Conceptualization, Data curation, Formal analysis, Visualization, Writing – original draft. TH: Data curation, Formal analysis, Writing – original draft. PP: Data curation, Formal analysis, Writing – original draft. C-LC: Data curation, Formal analysis, Writing – original draft. KS: Data curation, Formal analysis, Writing – original draft. PK: Conceptualization, Supervision, Validation, Writing – review & editing. EN: Conceptualization, Funding acquisition, Project administration, Supervision, Validation, Writing – original draft, Writing – review & editing.
