# Supplementary material for: Second bone marrow transplantation into regenerating hematopoiesis enhances reconstitution of immune system
Source: Front Immunol. 2024 Jun 14;15:1405210. doi: 10.3389/fimmu.2024.1405210 (PMC11211250; doi:10.3389/fimmu.2024.1405210)
Supplement: Supplementary file 1 [file Presentation_1.pdf]

Supplementary material

Second bone marrow transplantation into regenerating hematopoiesis enhances reconstitution of immune system

Kateřina Faltusová, Martin Báječný, Tomáš Heizer, Petr Páral, Chia-Ling Chen, Katarína Szikszai, Pavel Klener, Emanuel Nečas

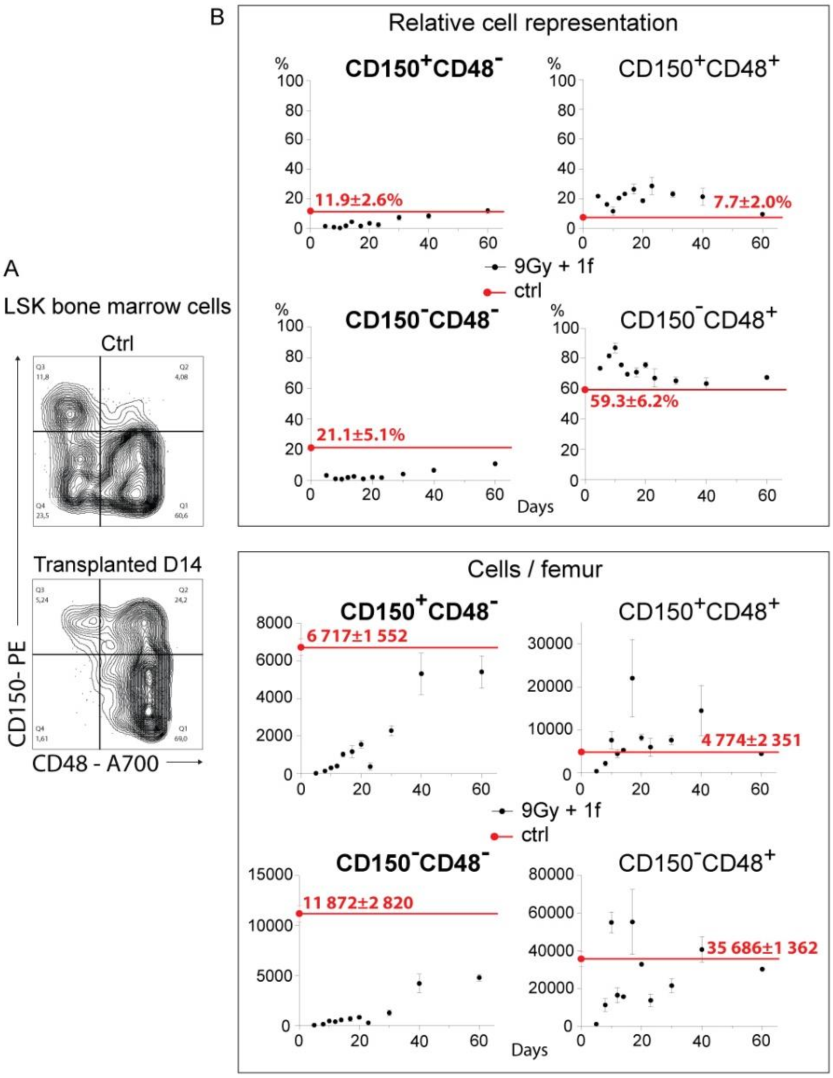

Supplementary Figure 1 CD48^{-} LSK cells are reduced during post-transplantation hematopoiesis reconstitution. Bone marrow cells of mice transplanted bone marrow cells from one femur (the peripheral blood, bone marrow cellularity, spleen weight and bone marrow reconstitution capacity of the mice are shown in Figure 1; main body) were stained with fluorescently labeled antibodies to determine lineage negative, Sca-1 positive, c-Kit positive (LSK) cells and their four subtypes characterized by the expression of CD48 and CD150 markers. (**A)** An example of CD48/CD150 plot of LSK cells in normal bone marrow (Ctrl) and bone marrow collected 14 days after transplantation (Transplanted D14). (**B)** Relative and absolute number of the four subtypes of LSK cells after transplantation. The red line shows values in the normal bone marrow. Data are means ± SEM (n=3).

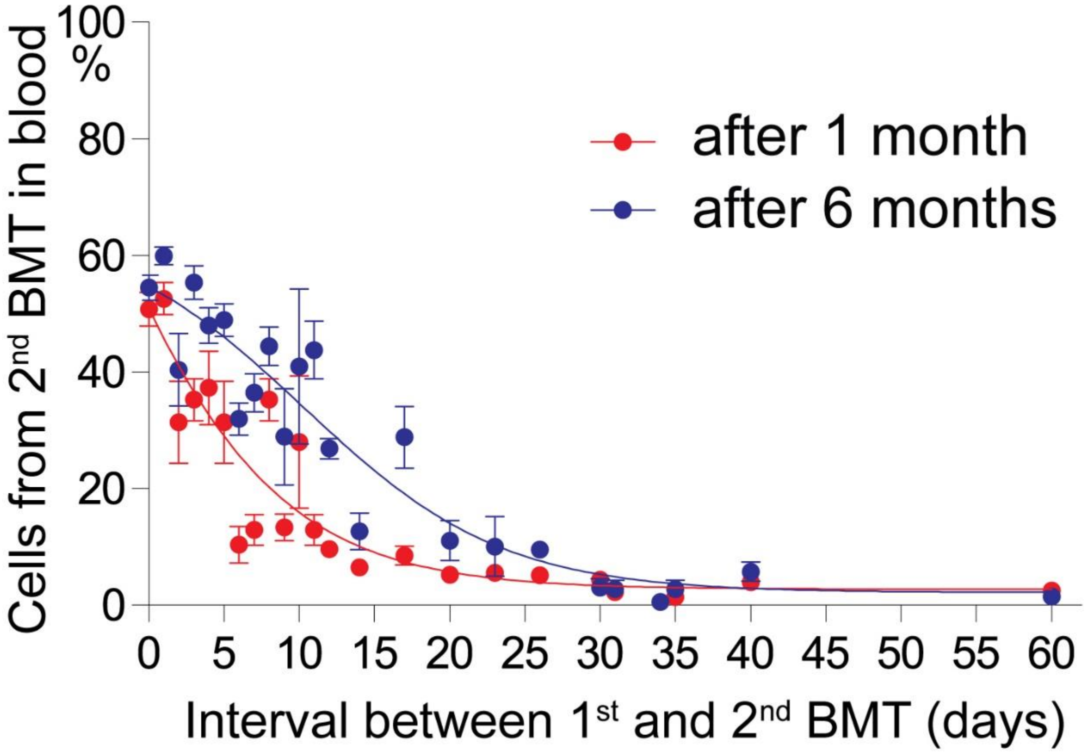

Supplementary Figure 2 Second BMT is more effective when its outcome is determined after six months (blue) than after one month (red). A nonlinear regression using the log (inhibitor) versus response curve - variable slope (4 parameters) calculation through data presented in Figure 2C (main body) without distinguishing individual experiments.

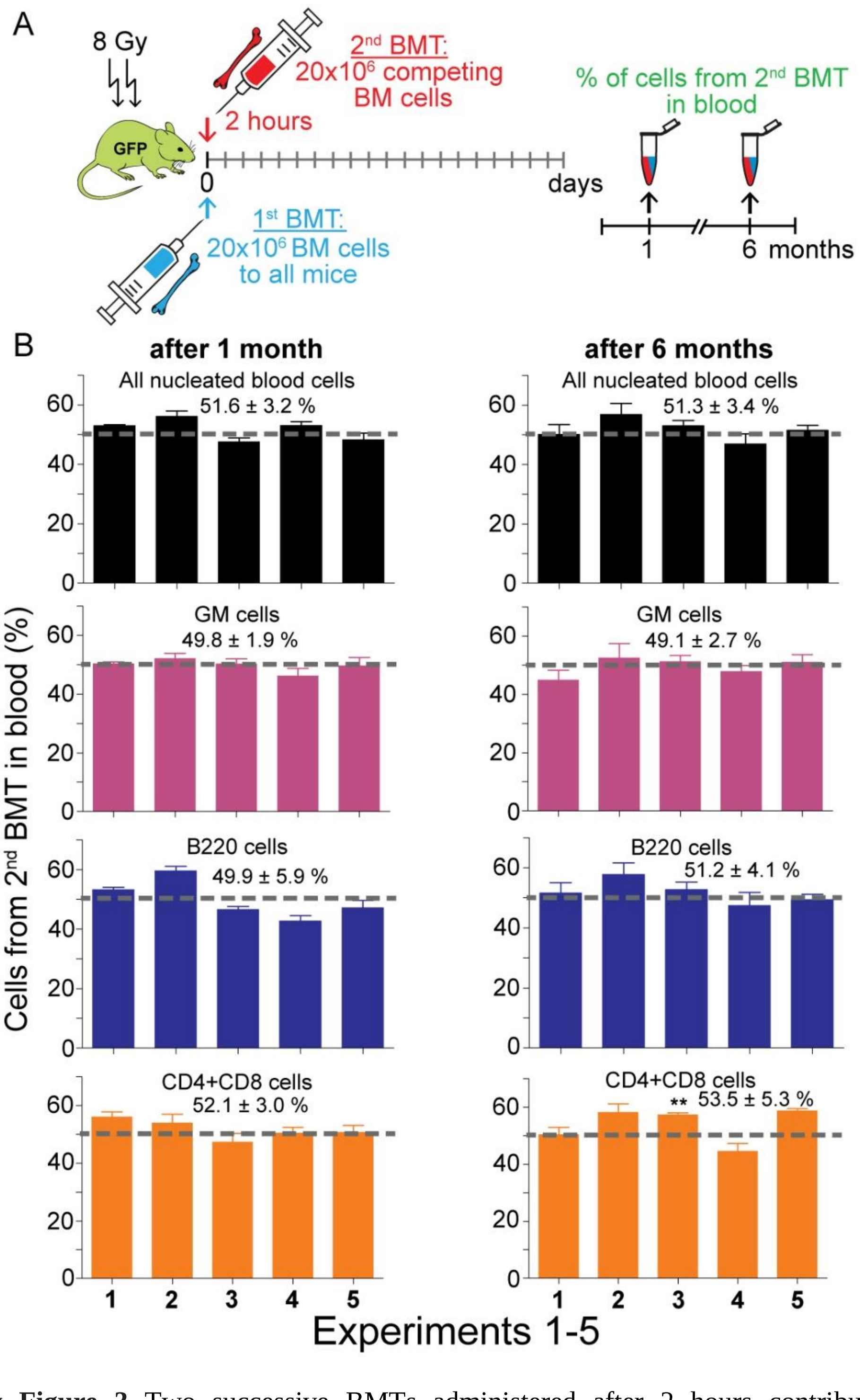

Supplementary Figure 3 Two successive BMTs administered after 2 hours contribute equally to reconstituted hematopoiesis. Results are from groups of mice given the 2nd BMT 2 hours after the 1st BMT in five independent experiments. **(A)** Experimental design. Irradiated UBC-GFP mice were treated by two successive BMTs of 20 million of bone marrow cells. Transplanted cells with different CD45.2 and CD45.1 marker. Details of the five experiments are in Table 1 (main body). A sample of blood was examined for the percentage of nucleated blood cells with the CD45 marker corresponding to the 2nd BMT after one and six months. (**B)** The blood nucleated cells were stained with fluorescently labeled antibodies to distinguish granulocytes and monocytes (GM), B cells (B220), and T cells (CD4+CD8). The equal contribution (50:50%) of both transplants to blood cell production is depicted by a dashed line. Data are means ± SEM. **p<0.01 against 50 %.

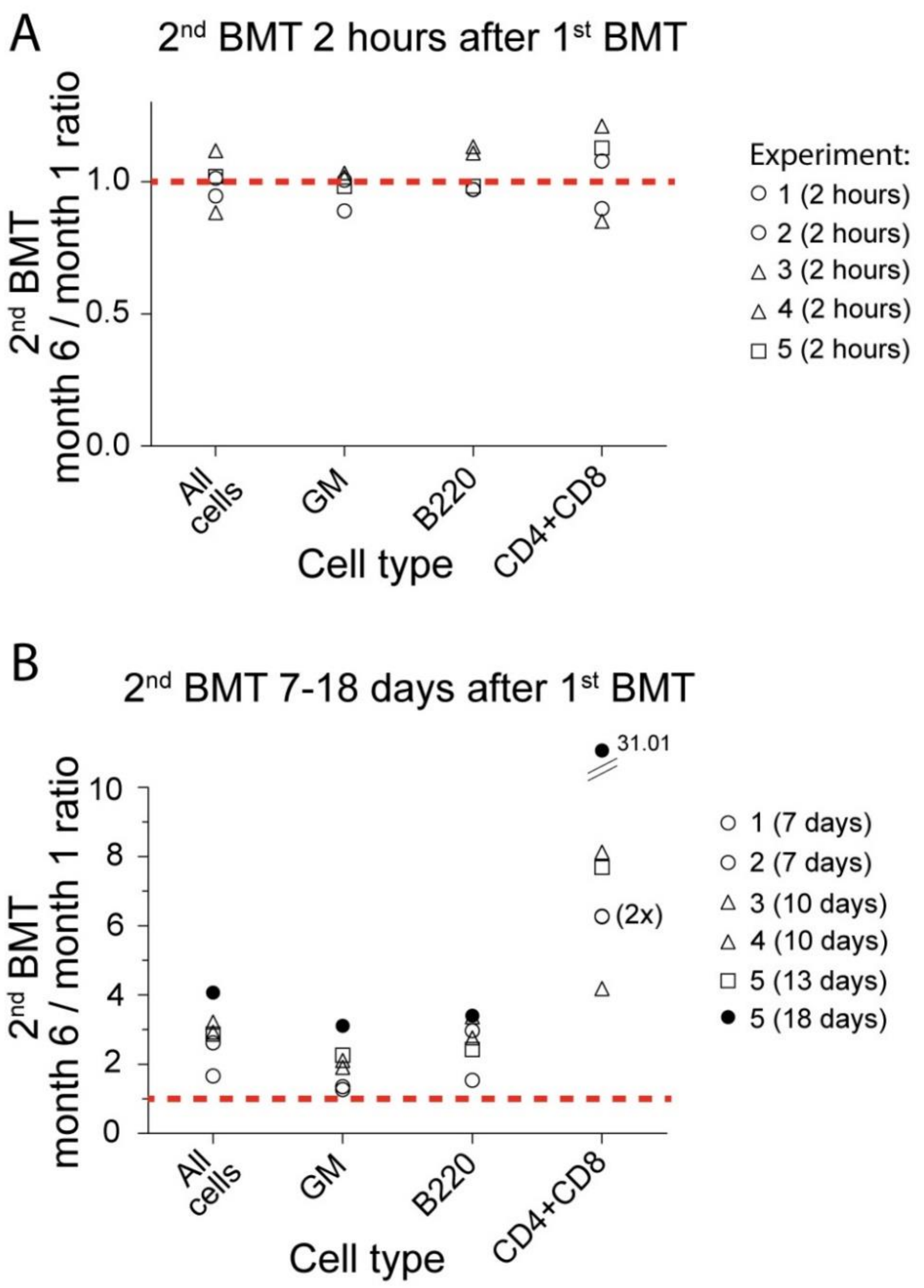

Supplementary Figure 4 Second BMT administered 7–18 days after the 1st BMT is more efficient when evaluated after 6 months than when evaluated after 1 month. The mean percentage of cells derived from the 2nd BMT determined after six months was divided by the percentage of the cells determined after one month (data are from Supplementary Fig. 3 and Figure 2). Details of the five experiments are in Table 1 (main body). The ratio between the six-month and one-month results are plotted separately for the groups of mice that received the second BMT 2 hours after the 1st BMT (**A**), and the groups of mice that received the 2nd BMT 7– 8 days after the 1st BMT (**B**)

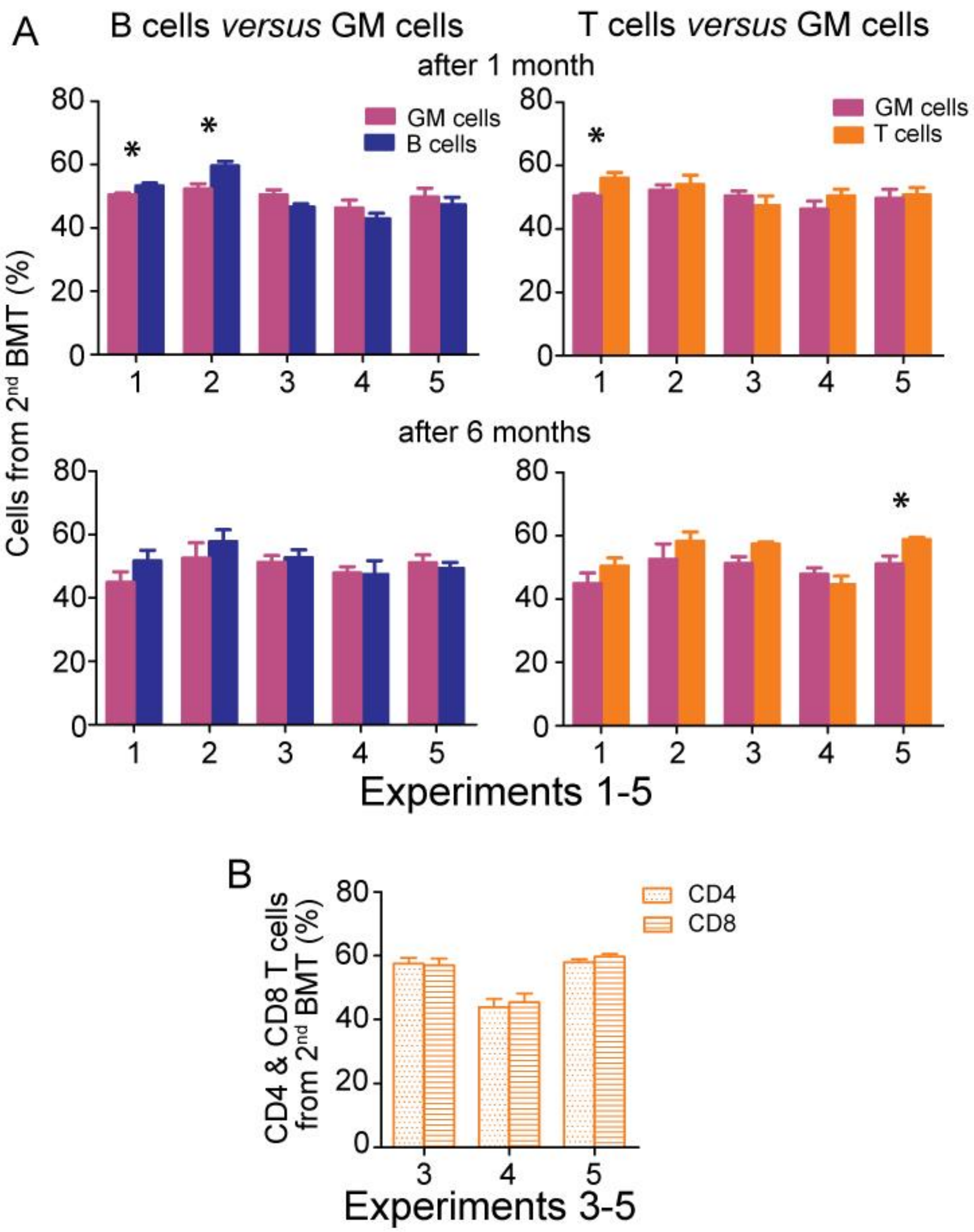

Supplementary Figure 5 Balanced production of lymphoid and myeloid cells from the 2nd BMT delivered 2 hours after the 1st BMT. **(A)** Frequency of B cells (B220) and T cells (CD4+CD8) derived from the 2nd BMT given 7 days (Experiments 1 and 2), 10 days (Experiments 3 and 4), 13 (empty circle) and 18 (full circle) days (Experiment 5) after the 1st BMT is compared to the frequency of granulocyte-monocytes (GM). Results are from the peripheral blood analyzed one and six months after the 2nd BMT. (**B)** In Experiments 3, 4, and 5, CD4 and CD8 T cells were distinguished six months after transplantation. * $p<0.05$.

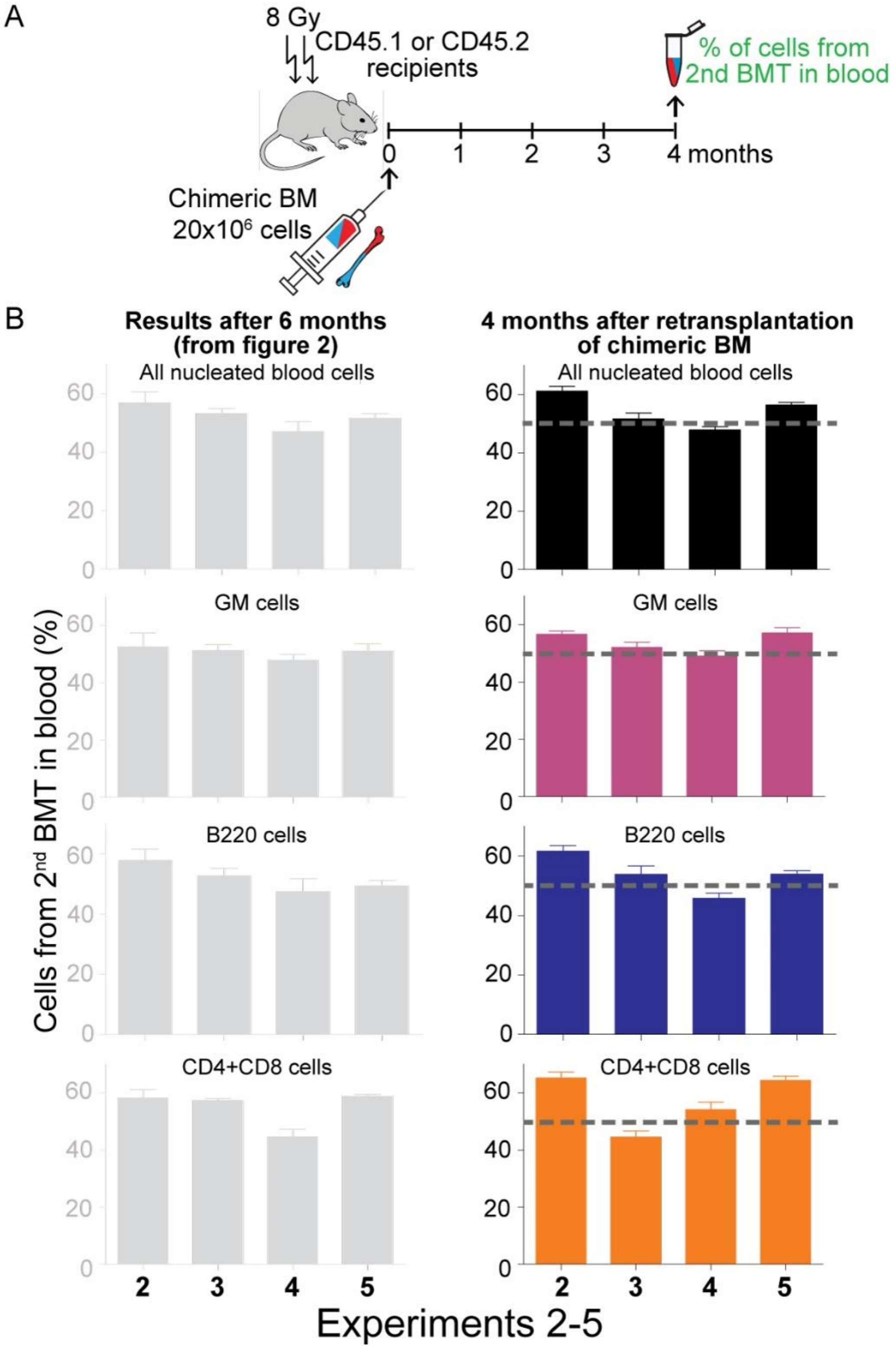

Supplementary Figure 6 Sustained production of blood cells from the 2nd BMT delivered 2 hours after the 1st BMT in secondary recipients. (**A)** Experimental design. Chimeric bone marrow was collected and pooled from groups of mice to which the 2nd BMT was given 2 hours after the 1st BMT. The bone marrow collected six months after the primary two successive BMTs was transplanted to secondary 8 Gy irradiated recipients (CD45.2 mice in Experiment 2; CD45.1 mice in Experiments 3, 4, and 5). Details of the five experiments are in Table 1 (main body). The peripheral blood cell was analyzed 4 months after chimeric bone marrow transplantation. Bone marrow from Experiment 5 was re-transplanted to the tertiary UBC-GFP recipients (see Supplementary Table 1). (**B)** The percentage of cells derived from the 2nd BMT is shown in the right column. For comparison, the corresponding results from the donors of chimeric bone marrow (presented previously in Supplementary Figure 3) are shown in the left column.

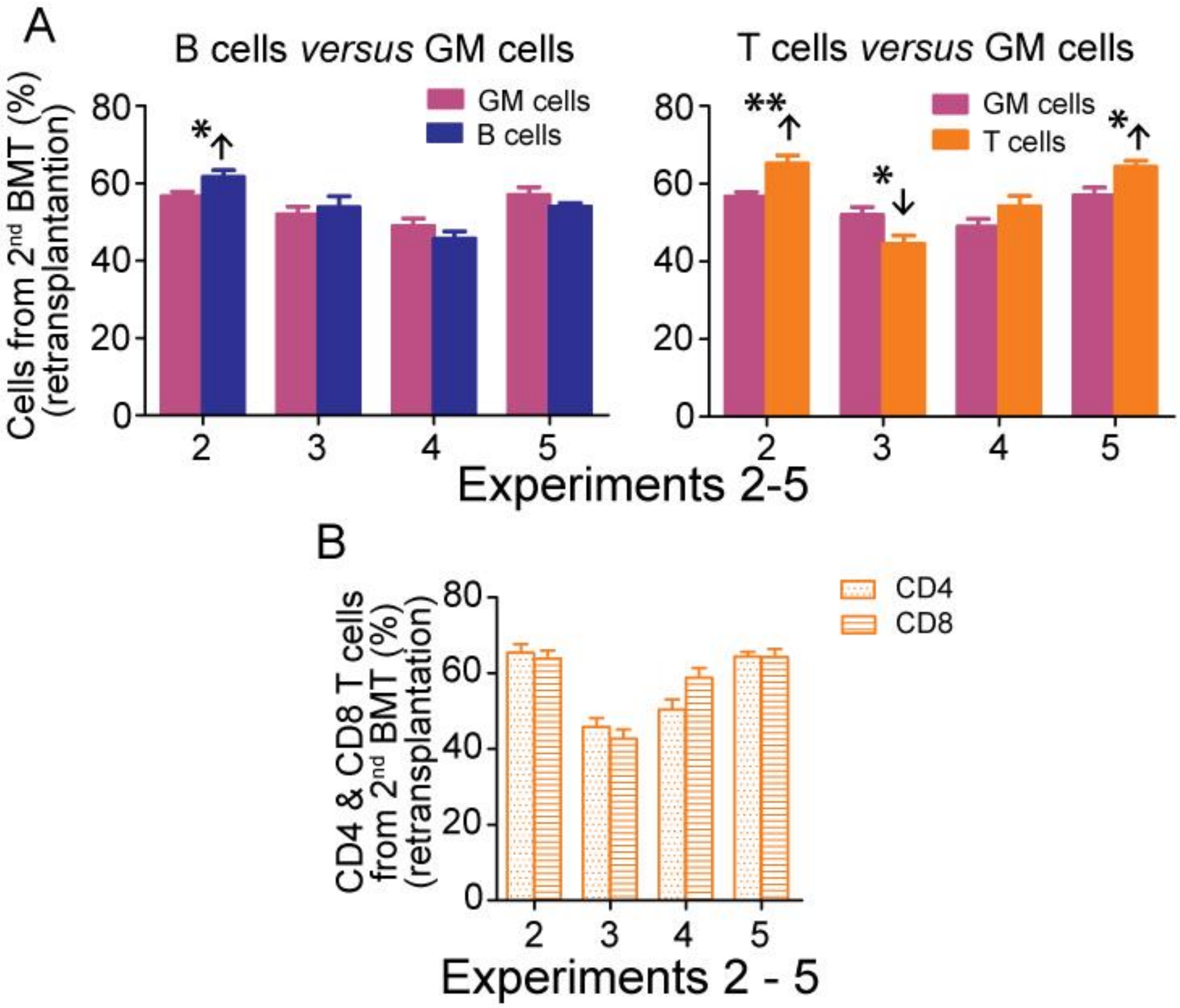

Supplementary Figure 7 Balanced production of myeloid cells and lymphoid cells from the 2nd BMT delivered 2 hours after the 1st BMT in secondary recipients. **(A)** Frequency of lymphoid B cells (B220) and T cells (CD4+CD8) derived from 2nd BMT delivered 7 days (Experiment 2), 10 days (Experiments 3 and 4), 13 (empty circle) and 18 (full circle) days (Experiment 5) after the 1st BMT is compared to the frequency of granulocytes and monocytes (GM) in the peripheral blood four months after chimeric bone marrow transplantation. (**B)** CD4 and CD8 T cells frequency in the peripheral blood. * $p<0.05$, ** $p<0.01$. Arrows indicate significantly increased or decreased values.

Supplementary Table 1

Experiment 5: Percentage of blood cells from 2nd BMT in 1st, 2nd, and 3rd recipients

D0

		All		GM		B220		CD4 + CD8		CD4		CD8	
recipients		**Mean**	**SEM**	**Mean**	**SEM**	**Mean**	**SEM**	**Mean**	**SEM**	**Mean**	**SEM**	**Mean**	**SEM**
**1st**	1 M	**49.24**	1.97	**50.17**	2.47	**47.38**	2.10	**55.55**	2.20	–	–	–	–
	4 M	**49.49**	1.77	**49.02**	1.25	**46.55**	2.25	**57.33**	0.96	**56.58**	1.13	**59.1**	0.81
	6 M	**55.08**	1.45	**51.08**	2.23	**49.37**	1.58	**58.79**	0.61	**57.98**	0.79	**51.08**	2.23
**2nd**	4 M	**56.4**	0.80	**57.06**	1.80	**53.94**	0.93	**64.35**	1.43	**64.39**	1.21	**64.29**	1.91
**3rd**	4 M	**57.47**	3.05	**62.1**	5.18	**53.99**	3.05	**63.55**	5.84	**61.94**	7.17	**65.86**	3.98
	6M	**59.65**	4.68	**65.66**	9.62	**54.78**	5.48	**62.55**	8.14	**63.09**	12.03	**62.19**	9.62

D13

		All		GM		B220		CD4 + CD8		CD4		CD8	
recipients		**Mean**	**SEM**	**Mean**	**SEM**	**Mean**	**SEM**	**Mean**	**SEM**	**Mean**	**SEM**	**Mean**	**SEM**
**1st**	1 M	**9.56**	0.45	**11.67**	0.73	**10.4**	0.49	**4.09**	0.36	–	–	–	–
	4 M	**27.92**	1.95	**26.81**	0.95	**25.86**	0.49	**30.27**	6.94	**35.52**	9.42	**20.68**	0.64
	6 M	**30.07**	0.82	**29.64**	0.55	**31.83**	1.33	**25.21**	0.48	**28.61**	0.35	**21.44**	0.92
**2nd**	4 M	**34.63**	0.95	**30.66**	2.33	**33.28**	1.17	**40.94**	2.59	**41.26**	2.45	**39.68**	3.39
**3rd**	4 M	**29.02**	5.23	**21.19**	1.99	**29.33**	5.85	**36.82**	11.23	**35.98**	12.03	**37.63**	10.04
	**6M**	**33.4**	6.1	**20.89**	5.6	**37.21**	14.52	**33.37**	14.82	**39.80**	11.06	**31.69**	5.59

D18

		All		GM		B220		CD4 + CD8		CD4		CD8	
recipients		**Mean**	**SEM**	**Mean**	**SEM**	**Mean**	**SEM**	**Mean**	**SEM**	**Mean**	**SEM**	**Mean**	**SEM**
**1st**	1 M	**5.92**	0.66	**8.06**	0.68	**7.01**	0.79	**0.79**	0.13	–	–	–	–
	4 M	**24.11**	2.52	**24.9**	3.14	**23.87**	1.98	**23.23**	4.81	**27.33**	6.65	**16.87**	1.57
	6 M	**34.86**	5.15	**28.85**	2.48	**34.41**	3.82	**34.73**	9.14	**40.39**	11.89	**19.25**	1.68
**2nd**	4 M	**39.95**	1.44	**33.05**	1.50	**42.13**	1.64	**40.63**	3.07	**42.58**	2.42	**37.38**	4.60
**3rd**	4 M	**43.87**	0.85	**36.2**	2.89	**46.94**	0.72	**45.94**	2.45	**44.14**	1.85	**47.94**	5.81
	6M	**38.45**	4.16	**35.25**	3.64	**42.05**	2.33	**32.75**	6.25	**33.17**	12.16	**38.52**	5.38

M - month(s)

starting results obtained 1 M after the 2nd BMT and shown in Figs. 3 & 5 and Ext. Data Figs. 3 & 6

results shown in Figs. 3 & 5 and Ext. Data Figs. 3& 6

results not shown in figures

Flow cytometry analysis of bone marrow and peripheral blood cells

Equipment and software

FACS Aria IIu flow cytometer equipped with 488 nm (50 mW), 561 nm (100 mW), 638 nm (140 mW), 405 nm (100 mW), and 355 nm (20 mW) lasers (BD Biosciences, San Jose, CA). The FACSDiva™ software (BD Biosciences, San Jose, CA), version 8.0.1, was used for data acquisition.

FACS Canto II flow cytometer, equipped with 405 nm (60 mW), 488 nm (20 mW), and 633 nm (15 mW) lasers (BD Biosciences, San Jose, CA). The FACSDiva™ software (BD Biosciences, San Jose, CA), version 6.1.3, was used for data acquisition. Data were analysed using FlowJo 10.8.2 (FlowJo, USA).

Data were analyzed by FlowJo 10.8.2 (FlowJo, USA).

Antibodies used for analysis of blood and bone marrow cells

Brilliant Violet 421™ **anti-mouse CD117 (c-Kit)** Antibody Clone 2B8 Cat. 105828 LOT: B299234 Biolegend 1:200 FC

Brilliant Violet 650™ **anti-mouse Ly-6A/E (Sca-1**) Antibody Clone D7 Cat. 108143 LOT: B326872 Biolegend 1:200 FC

Alexa Fluor® 700 **anti-mouse CD48** Antibody Clone HM48-1 Cat. 1117130 LOT: 179155 SONY 1:200 FC

PE **anti-mouse CD150** (SLAM) Antibody Clone TC15-12F12.2 Cat. 115904 LOT: B270365 Biolegend 1:200 FC

APC/Cyanine7 **anti-mouse TER-119**/Erythroid Cells Antibody Clone TER-119 Cat. 116223 LOT: B278991 Biolegend 1:200 FC

APC/Cyanine7 **anti-mouse/human CD11b** Antibody Clone M1/70 Cat. 101226 LOT: B242597 Biolegend 1:200 FC

APC/Cyanine7 **anti-mouse Ly-6G/Ly-6C (Gr-1)** Antibody Clone RB6-8C5 Cat. 557661 LOT: 9178588 BD Pharmingen 1:200 FC

APC/Cyanine7 **anti-mouse/human CD45R/B220** Antibody Clone RA3-6B2 Cat. 103224 LOT: B321245 Biolegend 1:200 FC

PE/Cyanine7 **anti-mouse CD45.2** Antibody Clone 104 Cat. 109830 LOT: B355882 Biolegend 1:200 FC

PE **anti-mouse CD45.1** Antibody Clone A20 Cat. 110708 LOT: B115504 Biolegend 1:200 FC

APC anti-mouse CD8a Clone53-6.7 Cat. 553035 LOT: 9156781 BD Pharmingen 1:200 FC

Alexa Fluor® 700 **anti-mouse/human CD45R/B220** Antibody Clone RA3-6B2 Cat. 103232 LOT: B364528 Biolegend 1:200 FC

Brilliant Violet 750™ **anti-mouse CD4** Antibody Clone GK1.5 Cat. 100467 LOT: B259413 Biolegend 1:200 FC

Brilliant Violet 421™ **anti-mouse Ly-6G/Ly-6C (Gr-1)** Antibody Clone RB6-8C5 Cat. 1142170 LOT: 168421 Sony 1:2000 FC

Brilliant Violet 421™ **anti-mouse/human CD11b** Antibody Clone M1/70 Cat. 101236 LOT: B360997 Biolegend 1:2000 FC
